# Comparative Carbon Footprint Study of Self‐Monitoring Vs. Continuous Monitoring of Blood Glucose

**DOI:** 10.1002/gch2.202500118

**Published:** 2025-07-25

**Authors:** Aida Hosseinian, Annika Johansson, Jaakko Karvonen, Ari Nissinen, Atte Pitkänen, Laura Sokka

**Affiliations:** ^1^ Finnish Environment Institute (SYKE) Latokartanonkaari 11 Helsinki 00790 Finland

**Keywords:** carbon footprint, diabetes, glucose monitoring, healthcare system, life cycle assessment

## Abstract

Climate change is an increasingly important problem, and efficient mitigation requires actions in all fields. While the impact of individual medical devices is small, the total impact of all the devices is large, and their use is also growing with the increasing elderly population. Therefore, it is urgent that this study improves knowledge of the impacts of the production and use of medical devices to find ways to decrease them. This study examines the carbon footprint of two prevalent blood glucose monitoring methods for diabetes management: self‐monitoring of blood glucose and continuous glucose monitoring systems. Using cradle‐to‐grave life cycle assessment, the carbon footprint of six different devices across both techniques is evaluated. Components of these devices are disassembled, weighed, and the different plastic parts are chemically analyzed using Fourier‐transform infrared spectroscopy (FTIR) to accurately quantify their material composition. The results of this study show that the carbon footprint of self‐monitoring devices is generally lower compared to continuous glucose monitoring devices, unless the testing frequency of the glucose level is higher than normal, or the device is used for shorter than average periods. The primary contributors to the carbon footprint of self‐monitoring devices are disposable strips and lancets. Regarding the continuous method, a major part of the carbon footprint is attributed to the plastic material and the instruction leaflet. This research provides important insights for product manufacturers, policymakers, healthcare providers, and individuals with diabetes, for more environmentally conscious choices in diabetes management technologies.

## Graphical abstract

A comparative carbon footprint analysis of glucose monitoring devices using life cycle assessment reveals environmental hotspots of continous and self‐monitoring methods. Emphasizing the need for sustainable design innovations and waste reduction strategies to align clinical benefits with global environmental sustainability goals in diabetes care.

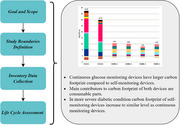

## 1 Introduction

The environmental impact of the healthcare system is a growing concern, as medical practices and technologies generate significant amounts of waste and consume a substantial quantity of resources.^[^ ^1^ ^]^ Approximately 4.6% of global greenhouse gas emissions are attributed to the healthcare system, and the share is expected to constantly increase, along with the growing economy and the ageing population.^[^ ^2^ ^]^ Conducting life cycle assessments for healthcare systems enables the evaluation of their environmental benefits and impacts, providing valuable support for eco‐friendly decision‐making. These assessments identify opportunities for resource efficiency, categorize and prioritize items according to their environmental impact, identify areas where development could yield the greatest environmental benefits, and promote further scientific research in the field.^[^ ^3^ ^]^

Among the different medical devices, glucose monitoring instruments used by individuals with diabetes represent a specific area of interest. Globally, there were 830 million people with diabetes in 2022, and this number is predicted to increase in the future.^[^ ^4^ ^]^ Therefore, addressing the carbon footprint of glucose monitoring devices is important as discussions on the sustainability of the healthcare system progress, while ensuring that individuals with diabetes receive effective care. Diabetes is a diverse group of chronic metabolic syndromes linked by a disorder in pancreatic function and elevated blood sugar.^[^ ^5^ ^]^ In this condition, it is essential for a person with diabetes to use medical devices to monitor their blood glucose levels and deliver the correct insulin dosage to maintain blood glucose balance.

There are several methods for measuring blood glucose. The most common method is testing blood samples or interstitial fluid with a glucose monitoring device.^[^ ^6^ ^]^ These devices can be divided into two types: self‐monitoring and continuous monitoring. For self‐monitoring of blood glucose (SMBG), the system includes a glucose meter, test strips, a lancet, and a lancet applicator. Self‐monitoring blood glucose meters are handheld devices that analyze a small drop of blood. A blood sample is typically taken from a fingertip with a lancet, after which the blood is made to interact with the test strip. Test strips, which are single‐use items, are inserted into the device for glucose measurement. Continuous glucose monitoring (CGM), on the other hand, consists of a sensor and a sensor applicator. In this method, a small sensor is attached to the skin that continuously measures glucose levels in the interstitial fluid via a needle, which penetrates the skin. The sensor uses a transmitter attached to the skin to send real‐time data to a display device or smartphone application. Each component of both techniques, including glucose meter, strips, lancets, sensor, and transmitter, plays a critical role in ensuring accurate, safe, and timely glucose monitoring, enabling effective diabetes management.

Glucose monitoring devices are produced by several manufacturers, each of them having their own specifications and designs. In order to accurately measure the carbon footprint of the two measurement practices, detailed data collection and analysis are needed at each life cycle stage. This involves using methodologies such as life cycle assessment (LCA), which enables the assessment of the environmental impacts of products or services from cradle to grave.

The life cycle of a glucose monitoring device begins with the extraction and processing of raw materials, such as metals, plastics, and electronic components, which require a significant amount of energy and resources. The manufacturing phase includes the assembly of devices and the production of consumables such as test strips and lancets, each contributing to climate impacts. Additionally, the way the products are used and their lifespan affect the amount of waste generated, which must be managed. The self‐monitoring process involves the frequent use and disposal of single‐use products, generating waste and associated emissions from production to disposal. However, the lifespan of the glucose meter is considerably longer (5–7 years). While components of continuous glucose monitoring, such as sensors and transmitters, have longer lifespans than single‐use strips, they can still only be used for 10–14 days. The frequent replacement of sensors, which are mainly manufactured from plastic and contain electronic components, causes an environmental burden in their production and end‐of‐life (EoL) phase.

This research enables a comparison of different glucose monitoring methods, identifying the factors that most significantly impact their carbon footprint. It can thus inform product development, research in healthcare sector, policymakers, healthcare providers, and patients, guiding them toward more environmentally friendly diabetes management options.

## 2 Methodology

Environmental impact assessments typically employ a methodical approach called life cycle assessment, which evaluates the environmental impacts associated with all stages of a product's life cycle. This begins with defining the goal and scope of the assessment, followed by setting up the system boundaries and determining which aspects of the product's life cycle will be considered. This is followed by data collection for inventory, which includes detailed information on energy consumption, material inputs, emissions, and waste generated at each stage. This comprehensive assessment helps to identify key areas for improvement, guides efforts to reduce environmental impacts, and develops more sustainable practices.

**Figure 1:**
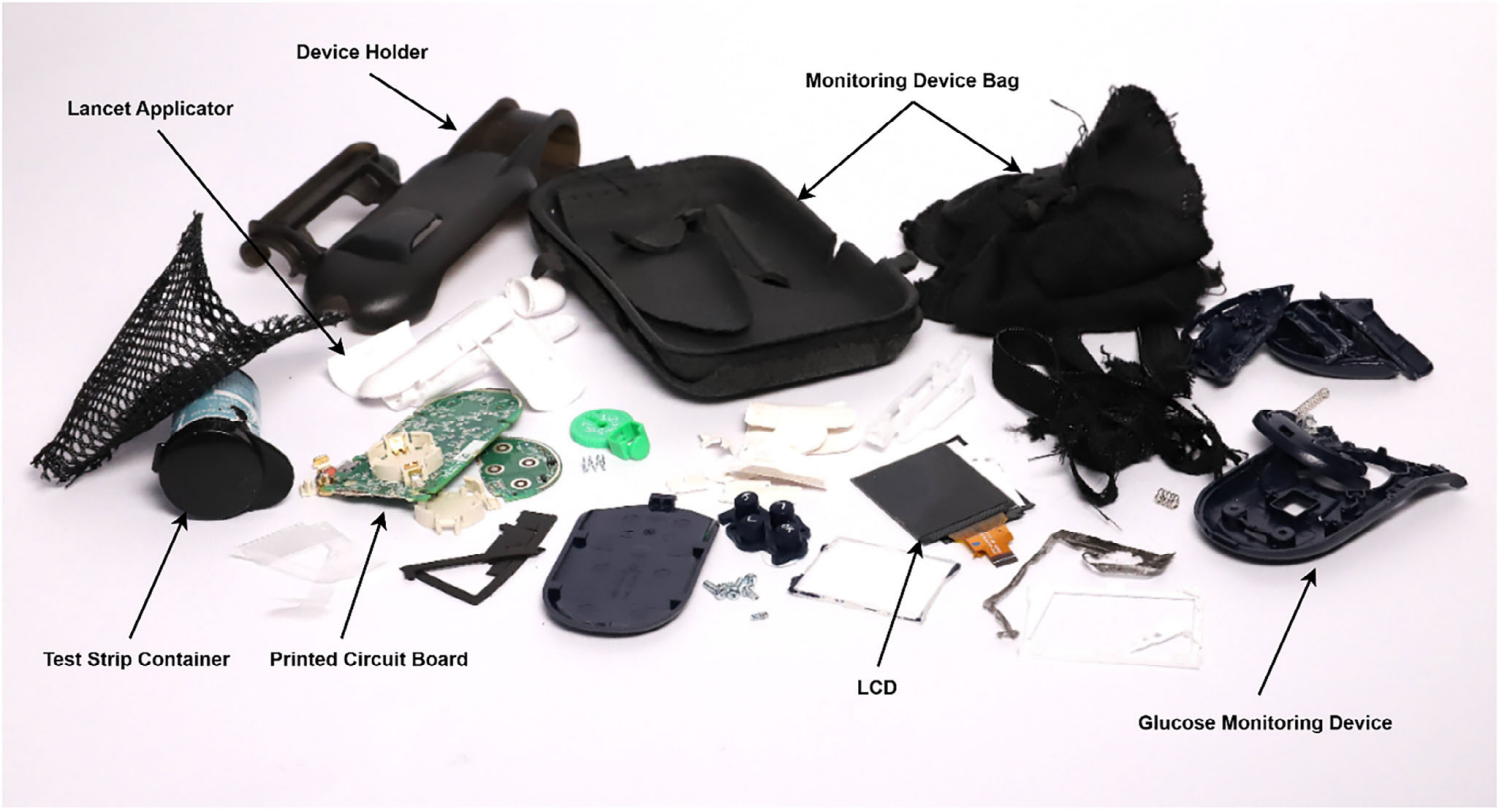
Dismantled continuous glucose monitoring device.

**Figure 2:**
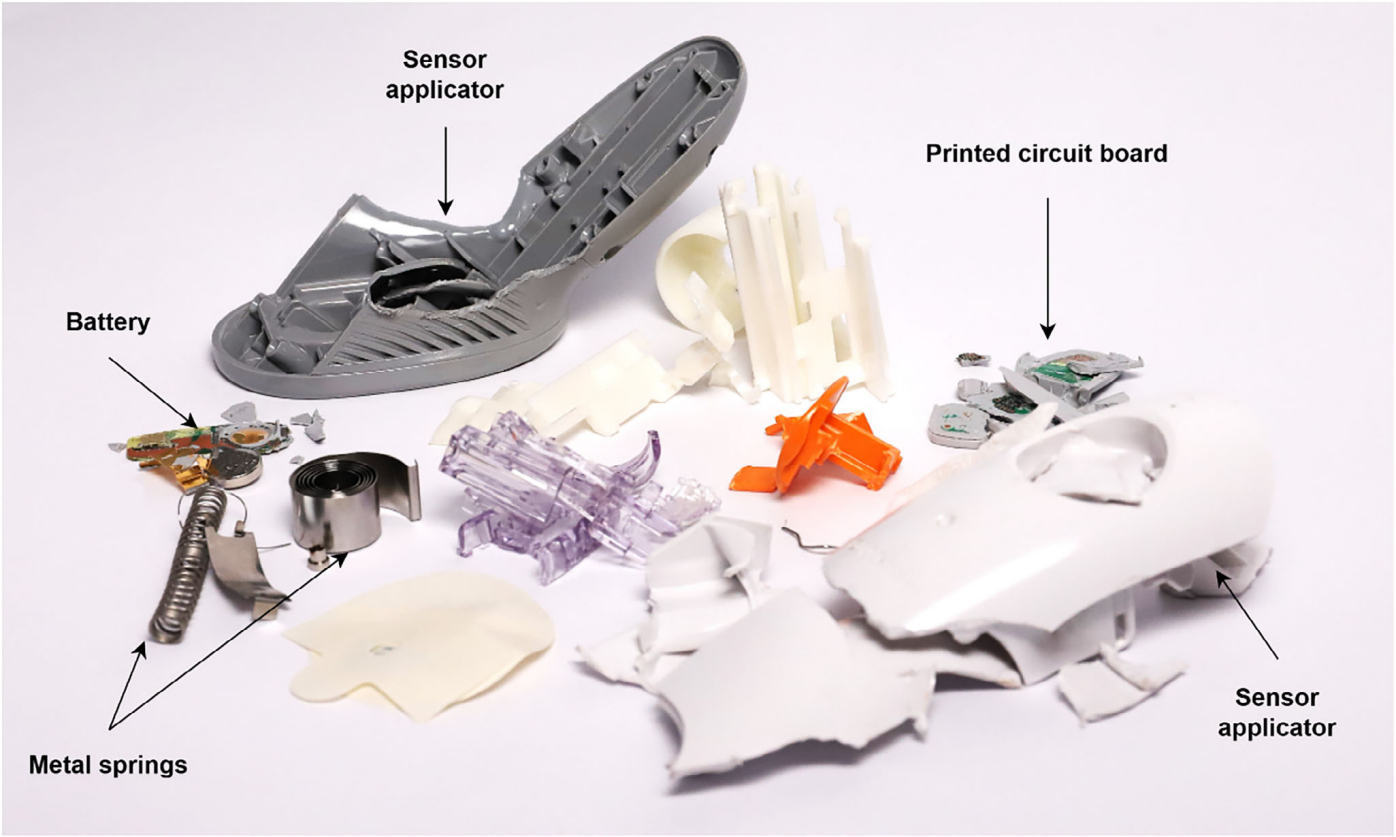
Disassembled self‐monitoring glucose measurement device.

To perform the life cycle assessment on the glucose measurement devices, each device was meticulously disassembled, weighed, and then the plastic components were subjected to chemical analysis. For the self‐monitoring devices, individual parts of the glucose meter, such as the plastic casing and printed circuit boards, were separated and analyzed. Additionally, the strips, lancets, lancet applicator, and device bag were disassembled and analyzed. Similarly, the continuous glucose monitors, including the sensor and transmitter, were taken apart to their constituent parts. The Fourier‐transform infrared spectroscopy (FTIR) chemical analysis method was used to identify plastics used in the manufacture of these components. This detailed breakdown provided a comprehensive understanding of the material composition and enabled the calculation of the climate impact associated with each part of the glucose monitoring devices. **Figure** **1** shows one device from the continuous glucose monitoring method, which was taken into pieces. Similarly, **Figure** **2** shows a self‐monitoring device that was disassembled for the material analysis process.

### 2.1 Description of the Glucose Monitoring Devices

In this study, six glucose monitoring devices were evaluated for their climate impacts: four self‐monitoring models that utilize disposable lancets and test strips, and two continuous glucose monitors designed to be worn on the body. The continuous monitoring devices are named CGM‐1 and CGM‐2, while the self‐monitoring devices are named SMBG‐1, SMBG‐2, SMBG‐3, and SMBG‐4. Detailed information on the lifespan and disposal practices, and a general description of each method has been compiled in **Table** **1**.

**Table 1:** Description of the glucose measurement devices.

Device		Lifespan	Disposal
Self‐monitoring glucose measurement (SMBG)	Glucose meter	5–7 years	Electrical waste recycling centers (WEEE), batteries to a recycling point
	Single‐use strips	Once, 5–10 used in a day	Incineration; mixed municipal solid waste
	Lancet	Once, 5–10 used in a day	Hazardous waste, Collection point in pharmacy (incineration)
	Lancet applicator	5–7 years	Incineration; mixed municipal solid waste
Continuous glucose monitoring (CGM)	Continuous glucose measurement device	10–14 days	Incineration; mixed municipal solid waste
	Sensor placement device	Once	Incineration; mixed municipal solid waste

The EoL phase considers the common practice of post‐consumer disposal in Europe. It was assumed that cardboard packaging, instruction leaflets, plastic containers of single‐use test strips, and electrical waste is sent for sorting and recycling in accordance with EU regulations (EU Directive on Packaging and Packaging Waste 94/62/EC, EU Directive on waste electrical and electronic equipment (WEEE) 2012/19/EU). The waste management of other components was determined using the diabetes care equipment sorting guide provided by the waste management company Vestia.^[^ ^7^ ^]^ It was assumed that the lancets are collected at pharmacies as hazardous waste, while the test strips are disposed of as mixed municipal solid waste, and it was anticipated that they would be sent for incineration with energy recovery. The environmental impacts of the treatment processes were retrieved from Ecoinvent v3.10.^[^ ^8^ ^]^

### 2.2 Life Cycle Assessment

This section outlines the LCA methodology used to estimate the carbon footprint associated with the glucose monitoring methods for their entire life cycle, in accordance with ISO 14040/14044 standards.^[^ ^9^, ^10^ ^]^ The LCA was conducted with SimaPro software (version 9.5.0.0).^[^ ^11^ ^]^ The global warming potential in midpoint impact categories of the ReCiPe hierarchical method have been used to assess the carbon footprint.^[^ ^12^ ^]^

#### 2.2.1 Goal and Scope

This study aims to compare the carbon footprint of two different methods for blood glucose monitoring, including self‐monitoring and continuous glucose monitoring systems with LCA. This LCA study is a cradle‐to‐grave assessment, including all life cycle stages from raw material extraction to the EoL stage of these devices. The carbon footprint was evaluated separately for both techniques used in the treatment of diabetes, and one year was used as the functional unit. The following parameters were defined based on actual usage patterns, product specifications provided in device manuals, and relevant regulatory frameworks. These reflect realistic conditions for glucose monitoring and form the basis of the life cycle assessment:

- The lifespan of CGM devices was set at 14 days, as specified by manufacturers, resulting in an annual use of 26 units per patient. One percent of the smartphone's carbon footprint was allocated to the use of the glucose monitoring application of the continuous monitoring method.
- The lifespan of SMBG devices was assumed to be seven years, consistent with typical durability and replacement cycles outlined in the product documentation. The use of lancets and test strips in the self‐monitoring method was set to an average of five per day (typical range 1‐10, depending on the type and state of diabetes).
- It was assumed that battery replacement for SMBG devices would occur after every 500 tests, as indicated in the device specifications and user manuals. The instruction leaflets were in two languages—Finnish and Swedish—in accordance with Finnish national legislation.
- EoL practices were based on national (Finnish) and EU waste management regulations.
- The delivery of products to customers was excluded from this study, as it was assumed to be the same for both glucose monitoring methods. However, the transport of materials within the production processes was included in the calculation, since “market activity” was used in the LCA.

#### 2.2.2 System Boundaries

**Figure 3:**
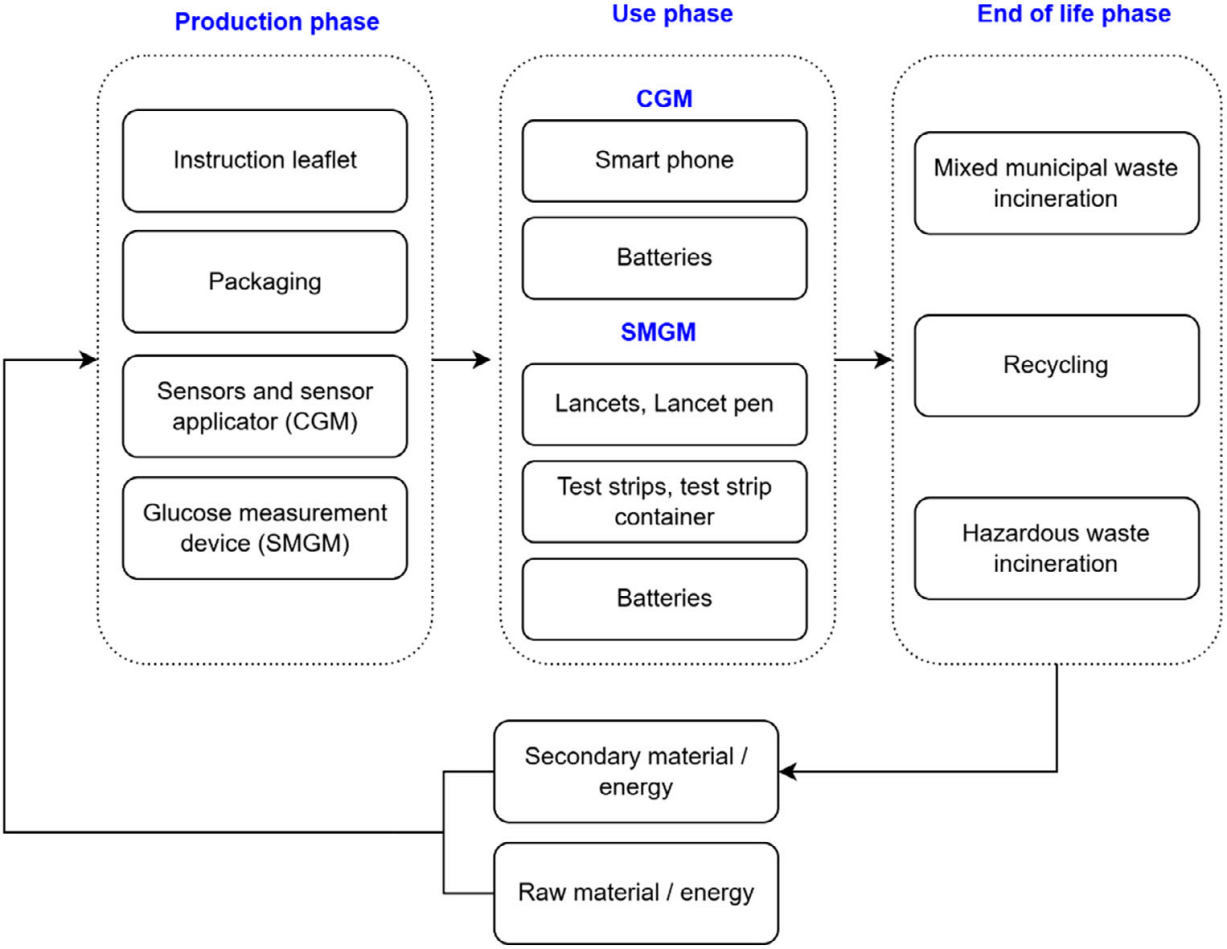
System boundaries of the study.

The system boundaries of the study are illustrated in **Figure** **3**. The system boundaries include production, use, and EoL phase of these devices, The study employs a system expansion approach, incorporating both the primary production and subsequent recycling scenarios whenever recycling is an option in the EoL phase.

#### 2.2.3 Life Cycle Inventory Analysis

The primary data of the foreground processes were obtained from the material analysis, device brochures, and the producers. The background processes were modeled using general data from the EcoInvent database version 3.10.^[^ ^13^ ^]^

#### 2.2.4 Uncertainty and Sensitivity Analysis

An uncertainty analysis was conducted to assess the reliability and robustness of the LCA results. Uncertainties were quantified using a Monte Carlo simulation technique, where multiple iterations were performed to simulate different possible outcomes based on variation in key parameters. The Monte Carlo simulation with 1,000 iterations was performed using Simapro software at a 95% confidence level. Additionally, a sensitivity analysis was applied to identify which inputs had the most significant impact on the results. Three factors were assessed:

1. Continuous and self‐monitoring devices lifespan.
2. Glucose level testing frequency in the self‐monitoring method.
3. Smartphone carbon footprint allocation to the glucose monitoring application in the continuous method.

This methodology ensured that the conclusions regarding the climate impacts of the devices were as accurate as possible, reducing uncertainty and reflecting the inherent variability in the data.

## 3 Results and Discussion

**Figure 4:**
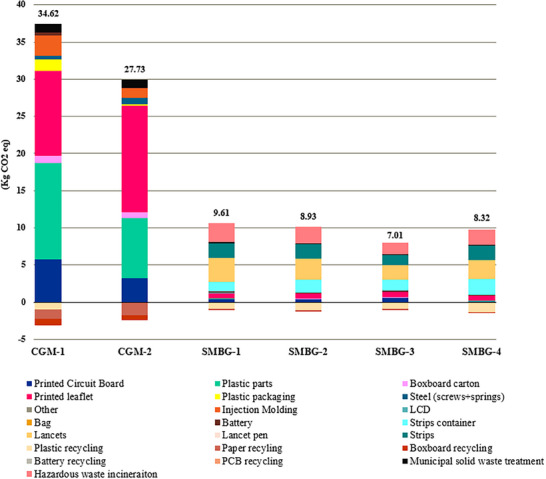
Carbon footprint of six devices: CGM 1, CGM 1, SMBG 1, SMBG 2, SMBG 3, and SMBG 4, representing contribution of the different components.

The carbon footprint of the six mentioned devices is presented in **Figure** **4**. The results show that CGM devices have a larger carbon footprint compared to SMBG devices. This difference is mainly attributed to material consumption, particularly the lifespan of single‐use equipment, which leads to higher consumption over the course of the year. In the case of SMBG devices, the components that require regular replacement are the lancets and test strips, which are lightweight and contribute to the lower carbon footprint than the CGM devices. Among the SMBG devices, the smallest carbon footprint belongs to device SMBG‐3, followed by SMBG‐4 and SMBG‐2, which are 7.01, 8.32, and 8.93 kg CO_2_ eq, respectively. The largest carbon footprint in this category is associated with device SMBG‐1 with 9.61 kg CO_2_ eq. For the continuous method, CGM‐1 has a larger carbon footprint with 34.62 kg CO_2_ eq compared to CGM‐2 with 27.73 kg CO_2_ eq. These rankings reflect the differences in materials used, expected lifetimes, and the overall design of each device.

As previously highlighted, the carbon footprint analysis of these devices shows that a notable part of the climate impact is primarily driven by the frequent replacement of essential single‐use components, such as sensors and applicators in continuous monitoring devices and lancets, and strips in self‐monitoring devices. A substantial portion of these components is made of plastics, which are the primary contributors to their carbon footprint. **Table** **2** shows the percentage of the three main contributors to the carbon footprint of each device.

**Table 2:** The primary contributors to the carbon footprint of glucose monitoring devices.

Primary contributor to the carbon footprint %						
	1st		2nd		3rd	
CGM‐1	36	Plastic parts	31	Instruction leaflet	16	Printed circuit boards
CGM‐2	49	Instruction leaflet	28	Plastic parts	11	Printed circuit boards
SMBG‐1	34	Lancets	27	Hazardous waste incineration (lancet waste)	20	Strips
SMBG‐2	32	Lancets	25	Hazardous waste incineration (lancet waste)	22	Strips
SMBG‐3	28	Lancets	25	Hazardous waste incineration (lancet waste)	20	Strips
SMBG‐4	31	Lancets	27	Hazardous waste incineration (lancet waste)	23	Strips

Regarding CGM‐1, the largest part of the carbon footprint is attributed to the regular replacement of sensors (plastic parts). These sensors typically need to be replaced every 10 to 14 days. Each sensor is applied to the body with an applicator, and both are discarded after the usage period to ensure safe use and proper functioning of the device. The need for frequent replacement increases the environmental impact, as the cumulative emissions from multiple sensor replacements over time lead to a substantial increase in the carbon footprint. In the case of the CGM‐1 device, 36% of the carbon footprint comes from the plastic components of the device's sensor and applicator.

Interestingly, for both the CGM‐1 and CGM‐2 devices, the paper leaflets make a major contribution to their carbon footprint. In fact, it was the largest contributor to the carbon footprint for CGM‐2. These leaflets weighed as much as 190 grams for CGM‐1 and 242 grams for CGM‐2, contributing 31% and 49% to these devices’ carbon footprints, respectively. Obligations regarding instructions for the use of medical devices come from Regulation 2017/745/ EU.^[^ ^14^ ^]^ The instruction leaflet must include information in the official languages of the member state, which in Finland, for example, means Finnish and Swedish. The regulation does not prohibit the use of additional languages; however, in these devices, two languages were included. Offering multilingual leaflets enables better product distribution, which is particularly important for items with expiration dates. As a result, the more languages included in the device instruction leaflet, the higher the weight and the CF. With the high volume of devices used each year, the accumulation of these paper leaflets results in a significant environmental impact. Given the global use of continuous glucose monitoring devices, the environmental burden associated with these instruction leaflets becomes a substantial factor in the total carbon footprint, even though their function is largely informational and likely important only for first‐time users of the devices. Therefore, the design of the leaflet could be optimized to reduce the device's carbon footprint. For example, the instructions could be provided in digital format while ensuring that the conditions specified in Regulation 207/2012/EU^[^ ^15^ ^]^ are fulfilled. Excluding the instruction leaflet from the total carbon footprint of CGM devices leads to a substantial reduction in their overall impact. For CGM‐1, the total carbon footprint would be 23.3 kg CO₂‐eq, with 55% attributed to plastic components. Under the same conditions, CGM‐2 would have a footprint of 13.33 kg CO₂‐eq, with plastics contributing 60% of the total. In both devices, the second‐largest contributor to the carbon footprint is the printed circuit board.

For SMBG devices, the carbon footprint is driven primarily by the consumables used in each measurement (Table 2). The most significant contributor to the environmental burden of these devices is the incineration of hazardous waste, which is the EoL phase for lancets. Lancets should be disposed of via hazardous waste incineration, as they may contain pathogens and biohazardous materials. Incineration is currently the most common practice and technology for the safe management and disposal of medical waste. On the other hand, this process generates emissions and environmental impacts due to the high temperatures used to destroy organic materials and sterilize waste.^[^ ^16^ ^]^

After hazardous waste incineration, lancets have the highest contribution to the carbon footprint in self‐monitoring glucose devices. Approximately 28–34% of the carbon footprint is attributed to the lancets, which are mainly produced from plastic material. Daily use of this component leads to a substantial total number of lancets used annually (five per day and 1825/annum). The third largest contributor to the carbon footprint in SMBG devices is test strips, which account for 20–23% of the total carbon footprint. Test strips are produced primarily from durable, flexible material like polyester or polycarbonate, and they contain an electrode, which is often silver or gold. Although the test strips are lightweight, as a result of their daily use a considerable amount of waste is generated over a year. It is also worth noting that while the silver makes up only 1% of the weight of the strips, it contributes nearly as much to the carbon footprint as the main body of the strips.

Another key factor to consider with regard to test strips is that they are typically packaged in plastic containers. Their frequent use results in more containers being discarded, significantly increasing the carbon footprint of the SMBG devices. In total, the carbon footprint of strips and their containers contributes to 33–48% of the carbon footprint of SMBG devices, which highlights how the design of these components could lead to significant reductions in the device's overall carbon footprint.

In the context of the EoL phase, results show that excluding this phase would have a significant impact on the devices’ overall carbon footprint. In the case of continuous monitoring devices, the recycling of paper and cardboard reduces the carbon footprint by ≈5% for CGM‐1 and CGM‐2. However, for SMBG devices, excluding the EoL phase would decrease their carbon footprint. This is due to the significant environmental impacts of incinerating lancets as hazardous waste. In both cases, these results highlight the importance of user behavior and recycling practices in determining the sustainability of different devices.

The effectiveness of recycling relies on national and EU regulations, the availability and practices of recovery and recycling facilities, as well as individual habits. These factors play a crucial role in reducing the environmental impact of glucose monitoring devices in use and EoL phases. For instance, plastic recycling, which is regulated by EU legislation (Directive 94/62/EC and 2018/852/EU),^[^ ^17^, ^18^ ^]^ can significantly lower the overall carbon footprint in LCA through system expansion. The system expansion approach is applied when recycled materials substitute virgin materials, resulting in credits for the system due to the avoided production of virgin material. In Finland, the primary part of the plastics in CGM devices are disposed of in mixed waste. However, the situation is different for SMBG devices. A significant part of the plastic material in these devices comes from the strip containers, which can be collected separately and recycled, thus helping to reduce the device's overall carbon footprint. However, plastic recycling practices may vary among different countries based on waste management strategy and the availability of facilities. This highlights how separate waste collection practices for recycling medical devices can have a meaningful impact on their sustainability. The results from Bassani et al. (2024)^[^ ^19^ ^]^ present a similar argument in terms of pharmaceutical packaging waste: environmental impacts in the EoL phase are strongly influenced by user behavior and the available treatment at a specific location. To enhance the environmental performance of pharmaceutical packaging, it is crucial to educate users through labeling, sorting instructions, and awareness campaigns. This should be combined with decreasing the amount of packaging, choosing materials with lower environmental impact, optimizing EoL valorization, and an effective waste management policy.

There are factors that cannot be incorporated into LCA studies. Therefore, to reach accurate conclusions on the topic, it is essential to consider other aspects of glucose monitoring systems in addition to the LCA results. For instance, it has been established that continuous glucose monitoring devices are the most accurate existing glucose management system for people with diabetes.^[^ ^20^ ^]^ These devices achieve continuous and real‐time results, making it possible to anticipate future glucose concentrations by tracking variations in glucose levels over time.^[^ ^21^ ^]^ Furthermore, these devices are easier and more convenient to use. However, the results from Procaccia et al. (2023)^[^ ^22^ ^]^ show that most diabetic individuals use the self‐monitoring system, which also costs less than the continuous method. However, self‐monitoring of blood glucose can have a disadvantage since it is perceived as painful and carries a risk of infection for diabetic individuals. This method only provides glucose levels at a specific moment, necessitating frequent pricks of the finger for more consistent monitoring, which increases the risk of infection. Other medical research results show that individuals who use self‐monitoring glucose measurements usually avoid checking their glucose levels due to several reasons, such as fear of losing their fingerprint or financial concerns.^[^ ^23^ ^]^ This highlights the importance of evaluating glucose monitoring devices from a broader perspective, including medical, financial, and psychological factors.

Furthermore, it should be acknowledged that environmental inventory data specific to medical‐grade materials are still under development, which may introduce some uncertainty into the life cycle assessment results presented in this study. In the absence of such data, this study has calculated the carbon footprint primarily through material analysis. While this approach introduces some limitations, it provides a robust basis for evaluating environmental impact. Given the high global prevalence of diabetes and the expected ongoing demand for glucose monitoring, the results presented here offer a valuable foundation for future innovations aimed at minimizing the environmental footprint of both SMBG and CGM technologies. One example of a data gap is the lack of information on the sterilization of specific components in glucose management devices.

For CGM devices, the needle and inserter must be sterilized, while in SMBGs, sterilization is required for the lancets. Sterilization is essential for ensuring the safety of these single‐use medical components, but it also introduces environmental impacts. Ethylene oxide is widely used for materials sensitive to heat or radiation, while gamma irradiation predominantly sourced from Cobalt‐60 is another common method. These processes are energy‐intensive and contribute to the carbon footprint of medical devices. Gamma radiation in particular has a considerable environmental burden, and ongoing challenges related to the supply of Cobalt‐60 have driven interest in alternative methods such as X‐ray and electron beam technologies.^[^ ^24^, ^25^ ^]^ Therefore, the actual carbon footprint of the CGM and SMBG devices evaluated in this study may be higher than calculated, as sterilization impacts were not included in the scope.

### 3.1 Sensitivity and Uncertainty Analysis

This section presents the results of the sensitivity and uncertainty analysis conducted for this LCA study. Sensitivity analysis was used to assess how variations in key input parameters, assumptions, and models impact the overall carbon footprint. The carbon footprint of diabetes management devices is primarily influenced by how frequently the diabetic individuals check their blood glucose levels and the device's lifespan. Furthermore, the analysis examined the impact of uncertainty on the assumptions regarding the carbon footprint allocation associated with using a smartphone glucose measurement application in the CGM method.

To assess the impact of these factors, the lifespan of the self‐monitoring device was adjusted from seven years to five, and the number of daily measurements was calculated for both five and ten times per day. As shown in **Table** **3**, the carbon footprint of SMBG devices can increase significantly with an increase in the number of daily measurements. However, reducing the lifespan of the monitoring device from seven to five years results in a slight increase in the carbon footprint of these devices. This is due to the environmental impact of the SMBG method being largely driven by the production and disposal of test strips and lancets. As the number of tests per day increases, both resource consumption and waste generation rise, which leads to substantial increase in the total carbon footprint.

**Table 3:** The impact of blood glucose measurement frequency and monitoring device lifespan on the carbon footprint in the self‐monitoring of blood glucose method.

Device's carbon footprint	Monitoring devices lifespan: 7 years Strip and lancet usage: 5 (baseline scenario)	Monitoring devices lifespan: 5 years Strip and lancet usage: 5	Monitoring devices lifespan: 5 years Strip and lancet usage: 10
SMBG‐1 (kg CO_2_ eq/year)	9.61	13.45	27.48
SMBG‐2 (kg CO_2_ eq/year)	8.93	12.50	25.86
SMBG‐3 (kg CO_2_ eq/year)	7.01	9.82	20.08
SMBG‐4 (kg CO_2_ eq/year)	8.32	11.65	24.86

**Table** **4** shows the results of the sensitivity analysis for the carbon footprint of CGM devices when the lifespan of the sensor is reduced to ten days. The results show that when the lifespan is reduced from 14 days to ten, the carbon footprint increases by about 30%. Comparing the results of SMBG devices, with higher tests per day and a shorter monitor lifespan, with the CGM with a 14‐day lifespan, an interesting observation is revealed. When self‐monitoring devices are used for shorter periods and with more daily measurements, the difference in carbon footprint between these two types of devices becomes smaller. This suggests that if the monitoring device of the SMBG method is replaced with a new one after a shorter period and tests are done more often per day (in a more severe diabetic condition), CGM and SMBG devices have a similar environmental impact.

**Table 4:** The impact of lifespan on the carbon footprint of the continuous glucose monitoring method.

Device	Lifespan 14 days (baseline scenario)	Lifespan 10 days
CGM‐1 (kg CO_2_ eq/year)	34.62	49.17
CGM‐2 (kg CO_2_ eq/year)	27.73	39.36

**Table 5:** Change of the carbon footprint allocation associated with using a smartphone glucose measurement application in the continuous glucose monitoring method.

Device	1% (baseline scenario)	5%	10%
CGM‐1 (kg CO_2_ eq/year)	34.62	35.58	36.78
CGM‐2 (kg CO_2_ eq/year)	27.73	28.69	29.89

The data for the carbon footprint of smartphones has been retrieved from the Belkhir and Elmeligi (2018) study.^[^ ^26^ ^]^ For this study, for the baseline scenario for CGM devices, 1% of the use‐phase carbon footprint of a smartphone is allocated to the glucose monitoring application. To explore how changes in this assumption affect the result of the CGM method, two different allocation rates (5% and 10%) were examined. As can be seen in **Table** 5, adjusting the smartphone carbon footprint allocation assumption from 1% to 5% or 10% does not notably affect the carbon footprint of CGM devices. This suggests that this factor does not have a strong effect on the overall outcome, and the results are reliable under a range of assumption values.

**Table 6:** Uncertainty analysis of the global warming impact category using Monte Carlo method (SD: standard deviation; CV: coefficient of variation; SEM: standard error of mean).

	Unit	Mean	Median	SD	SEM	CV (%)
CGM‐1	kg CO_2_ eq/year	34.65	34.08	3.87	0.12	11.17
CGM‐2	kg CO_2_ eq/year	27.50	27.35	1.94	0.06	7.04
SMBG‐1	kg CO_2_ eq/year	9.61	9.55	0.96	0.03	9.98
SMBG‐2	kg CO_2_ eq/year	8.93	8.85	0.96	0.03	9.12
SMBG‐3	kg CO_2_ eq/year	6.99	6.89	0.63	0.02	9.12
SMBG‐4	kg CO_2_ eq/year	21.40	21.24	1.99	0.06	9.31

The results of the uncertainty analysis of the global warming impact category of the midpoint assessment method are reported in Table 6 based on the mean, median, standard deviation (SD), coefficient of variation (CV), and standard error of mean (SEM). As can be seen in the uncertainty results, values for CV% suggest that the results are consistent and reliable. This implies that the data shows little variation, which means that the carbon footprint remains consistent across different scenarios or assumptions. Among the six devices, CGM‐1 and SMBG‐4 have higher CV, which shows a bit more variability in the results. However, the values are within a moderate range, and they do not show high uncertainty. In general, all SEM values are relatively low, which indicates high precision in the mean value and shows the consistency of the results.

## 4 Conclusion

In conclusion, the carbon footprint analysis of the six different blood glucose monitoring devices, including both the self‐monitoring and the continuous monitoring method, reveals that continuous glucose monitoring devices generally have a greater carbon footprint than self‐monitoring devices. The main contributors to the carbon footprint of continuous monitoring devices were identified as plastic components and the paper instruction leaflets. Considering this, redesigning the sensor applicator to be reusable or to use less material, along with minimizing or digitizing user manuals, could significantly reduce their carbon footprint. In contrast, in self‐monitoring devices, lancets and strips were the primary contributors to the carbon footprint. Notably, the results also indicate that if the lifespan of a self‐monitoring device is shorter and the frequency of strip and lancet use is higher, the carbon footprint of self‐monitoring devices can increase to the same level as continuous devices. This finding underscores the importance of usage patterns and device lifespan in determining the environmental impact, suggesting that under certain conditions self‐monitoring devices can be just as environmentally intensive as continuous monitoring devices.

Despite the inherent limitations of life cycle assessment studies, such as their inability to account for factors like user comfort, convenience, or the need for real‐time monitoring, the insights they provide into the environmental impact of these devices are invaluable. As the reliance on medical devices continues to grow and the urgency of addressing climate change intensifies, these findings are crucial for developing effective strategies to reduce their environmental footprint.

## Conflict of Interest

The authors declare no conflict of interest.

## Author Contributions

A.H. performed conceptualization, data curation, formal analysis, methodology, software, validation, visualization, and writing—original draft. A.J. performed conceptualization, data curation, formal analysis, methodology, validation, visualization, writing—review, and editing. J.K. performed conceptualization, data curation, formal analysis, methodology, validation, visualization, writing—review, and editing. A.N. performed funding acquisition, project administration, supervision, resources, conceptualization, data curation, formal analysis, methodology, validation, visualization, writing—review, and editing. A.P. performed conceptualization, data curation, formal analysis, methodology, validation, visualization, writing—review, and editing. L.S. performed conceptualization, data curation, formal analysis, methodology, validation, visualization, writing—review, and editing.

## Data Availability

The data that support the findings of this study are available from the corresponding author upon reasonable request.
